# A derivative of platelet-derived growth factor receptor alpha binds to the trimer of human cytomegalovirus and inhibits entry into fibroblasts and endothelial cells

**DOI:** 10.1371/journal.ppat.1006273

**Published:** 2017-04-12

**Authors:** Cora Stegmann, Daniel Hochdorfer, Diana Lieber, Narmadha Subramanian, Dagmar Stöhr, Kerstin Laib Sampaio, Christian Sinzger

**Affiliations:** Institute of Virology, University of Ulm, Ulm, Germany; Louisiana State University Health Sciences Center, UNITED STATES

## Abstract

Human cytomegalovirus (HCMV) is a widely distributed herpesvirus that causes significant morbidity in immunocompromised hosts. Inhibitors of viral DNA replication are available, but adverse effects limit their use. Alternative antiviral strategies may include inhibition of entry. We show that soluble derivatives of the platelet-derived growth factor receptor alpha (PDGFR-alpha), a putative receptor of HCMV, can inhibit HCMV infection of various cell types. A PDGFR-alpha-Fc fusion protein binds to and neutralizes cell-free virus particles at an EC50 of 10–30 ng/ml. Treatment of particles reduced both attachment to and fusion with cells. In line with the latter, PDGFR-alpha-Fc was also effective when applied postattachment. A peptide scan of the extracellular domain of PDGFR-alpha identified a 40mer peptide that inhibits infection at an EC50 of 1–2 nmol/ml. Both, peptide and fusion protein, were effective against various HCMV strains and are hence promising candidates for the development of novel anti-HCMV therapies.

## Author summary

Human cytomegalovirus (HCMV) depends on expression of platelet-derived growth factor receptor alpha (PDGFR-alpha) for infection of fibroblasts whereas this cell surface protein is not required for infection of endothelial cells. Surprisingly, pretreatment of HCMV with a soluble derivative of PDGFR-alpha prevents infection of both cell types, most probably via specific binding to the trimeric gH/gL/pUL74 complex. While adsorption is inhibited in both cell types, an additional penetration block occurs only in fibroblasts. The finding that an essential molecular interaction of HCMV with fibroblasts can be subverted for inhibition of the virus provides an antiviral strategy that may be hard to circumvent by the virus.

## Introduction

Human cytomegalovirus (HCMV) is a pathogenic human beta-herpesvirus that shares the property of other beta-herpesviruses to replicate only in its specific host. Primary infection is followed by lifelong latent persistence with occasional reactivation of the virus, which usually goes unnoticed by the infected individual. However, under conditions of insufficient immune responses, HCMV can cause severe or even life-threatening disease, e.g. in AIDS patients, transplant recipients, and fetuses infected *in utero*. Although antiviral drugs are available, their use is limited due to associated adverse effects and the development of resistance [1, 2]. Therefore, alternative treatment options are desired.

One powerful antiviral strategy is the inhibition of entry into the host cell, as exemplified by the effective neutralizing activity of anti-HCMV antibodies [3–10]. While the therapeutic use of antibodies may be limited as they are difficult to engineer, other entry inhibitors are also conceivable for HCMV. Small molecules and peptides have already been approved for antiretroviral therapy [11], and a peptide-based entry inhibitor against Hepatitis B virus is in clinical trial [12]. In the case of picornaviruses, an Fc-CAR fusion protein inhibits viral entry and is effective in animal models, but has not yet been developed for clinical use [13–15].

HCMV is an enveloped virus and thus requires membrane fusion with the host cell to deliver its nucleocapsid into the cytoplasm. Several glycoprotein complexes in the envelope of HCMV particles have been described to contribute to the entry of HCMV into its target cells and are therefore potential targets for entry inhibitors [16–21]. By analogy with other herpesviruses, homotrimers of glycoprotein B (gB) are assumed to exert the fusion between viral envelope and cellular membrane, while heterotrimers of gH, gL and pUL74 (gO) are necessary to promote this fusion process [22–26]. On certain cell types like endothelial and epithelial cells, an additional pentameric complex is required for effective entry, which consists of gH, gL, and three accessory proteins from the viral UL128 gene locus [26–30].

On the cellular side, numerous proteins were proposed as entry receptors of HCMV, but have been controversially discussed: e. g. the epithelial growth factor receptor (EGFR) and the platelet-derived growth factor receptor alpha (PDGFR-alpha) [31–36] (reviewed in [28]).

Here, we show that the extracellular part of PDGFR-alpha is a highly potent entry inhibitor of HCMV in fibroblasts and endothelial cells, which represent the pentamer-independent and the pentamer-dependent entry pathway, respectively. PDGFR-alpha-derived small peptides are also effective, thus providing a rationale for the development of PDGFR-alpha based anti-HCMV therapeutics.

## Results

### Knockdown of PDGFR-alpha prevents HCMV infection of fibroblasts but not endothelial cells

Two cellular growth factor receptors, PDGFR-alpha and EGFR have been reported to promote HCMV infection in fibroblasts [31, 34]. However, their relevance for HCMV infection was questioned in subsequent studies [32, 36]. With regard to PDGFR-alpha, a very recent report confirmed its significance for HCMV infection [37]. As we aimed at exploring the potential of these molecules to serve as a basis for the development of HCMV entry inhibitors, the first step was to confirm their contribution to HCMV infection. To address the diverse entry pathways of HCMV, we applied a virus strain expressing both gH/gL complexes to infect two model cell types representing the restricted tropism (fibroblasts) and the extended tropism (endothelial cells).

**Fig 1:**
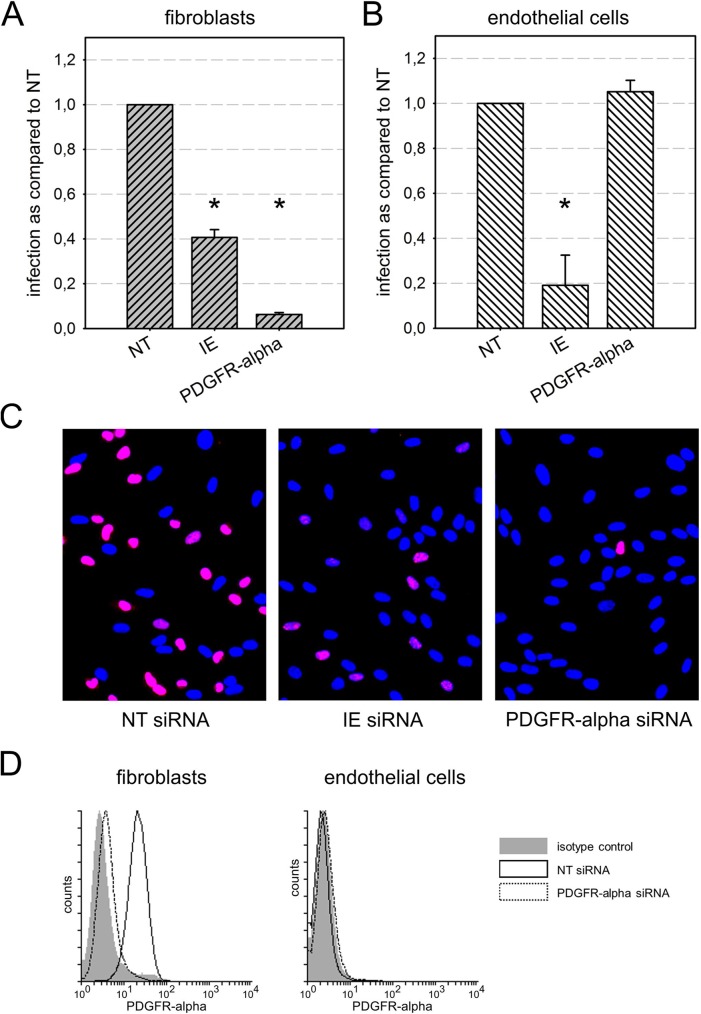
Effect of siRNA-mediated depletion of PDGFR-alpha on infection efficiency in fibroblasts and endothelial cells. Fibroblasts **(A)** and endothelial cells **(B)** were transfected with siRNAs targeting PDGFR-alpha. Non-targeting (NT) siRNAs and siRNAs against the viral immediate early (IE) proteins were included as controls. Two days after transfection, cells were infected with HCMV strain TB40/E, and the next day viral IE antigens were detected by indirect immunofluorescence for visualization of infected cells. The number of IE antigen-positive cells was counted and compared to the NT control. Error bars in (A) and (B) represent the standard error of the mean (SEM). Significant differences as compared to NT control are indicated by asterisks. **C**: Examples of HCMV IE antigen expression (red fluorescence) in HFFs after treatment with the respective siRNAs. Nuclei of non-infected cells appear blue due to counterstaining with DAPI. **D:** Fluorescence-activated cell sorting (FACS) for detection of PDGFR-alpha on the surface of fibroblasts and endothelial cells 2 d after treatment with PDGFR-alpha siRNA or non-targeting (NT) siRNA. NT siRNA represents PDGFR-alpha levels without specific knockdown. Isotype control antibody was included as a negative control for staining.

Using an siRNA approach, the respective growth factor receptors were targeted in human foreskin fibroblasts (HFFs) and endothelial hybrid cells (EA.hy926) two days before infection with HCMV strain TB40/E at a multiplicity of infection (MOI) of 1 infectious unit per cell, corresponding to about 60% infection. Cells treated with non-targeting siRNAs served as negative controls while an siRNA targeting viral immediate-early (IE) transcripts was included as a positive control. One day after infection, cell cultures were fixed and viral IE antigens were immunostained to determine the fraction of infected cells. In each of three experiments, the degree of infection obtained with the various treatments was normalized to the non-targeting control. As expected, the IE siRNA partially reduced the infection efficiency (by 60% in HFFs and 80% in endothelial cells, p-values > 0.01). Treatment with PDGFR-alpha siRNA almost completely prevented HCMV infection of fibroblasts (95% reduction; highly significant with a p-value < 0.001) whereas it had no inhibitory effect in endothelial cells (Fig 1). In line with these results, PDGFR-alpha was only found on the surface of HFFs but not of endothelial cells in FACS analyses, and surface expression in HFFs was substantially suppressed upon transfection with PDGFR-alpha siRNA (Fig 1D; for immunofluorescence stainings see S1 Fig, panel A).

In contrast, no EGFR-specific signal was detected on fibroblasts, and EA.hy926 cells had only a very weak signal. Treatment with EGFR-specific siRNA did neither reduce the amount of EGFR detectable on the cell surface (S1 Fig, panel B) nor reduce infection efficiencies in these cell cultures (S1 Fig, panel C). Hence, no conclusion on the role of EGFR for HCMV-infection was possible in our cell culture systems.

In summary, of the two growth factor receptor molecules that had previously been reported to promote HCMV entry, only the contribution of PDGFR-alpha was confirmed in our experimental setting.

### Pretreatment of HCMV with a soluble PDGFR-alpha-Fc chimera inhibits infection of fibroblasts and endothelial cells

**Fig 2:**
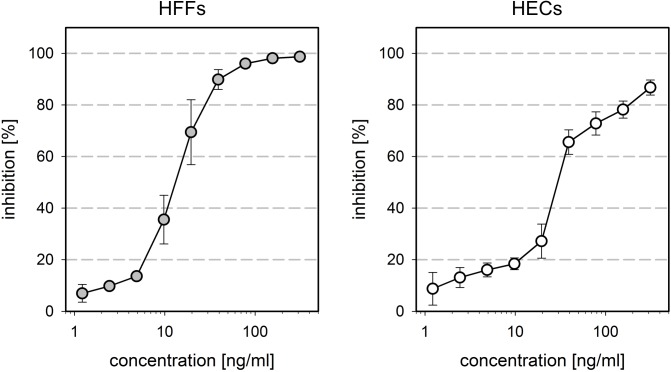
Inhibitory effect of soluble PDGFR-alpha-Fc chimeras on HCMV infection. Virus preparations of strain TB40/E were pretreated for two hours with PDGFR-alpha-Fc at indicated concentrations and then added to fibroblasts (HFFs) and endothelial cells (HECs). One day after infection, cells were fixed and stained for viral IE antigens by indirect immunofluorescence. The ratio of IE antigen-positive cells per total cell number was calculated to represent the degree of infection. The graphs show the inhibition of infection in treated cultures as compared to untreated controls. Error bars represent the standard error of the mean (SEM).

The strong dependence of HCMV infection on expression of PDGFR-alpha suggested an interaction between this cellular growth factor receptor and HCMV particles during the entry process in HFFs. Therefore, we hypothesized that pretreatment of viral particles with soluble forms of this cellular molecule might block the putative interaction sites on the surface of HCMV virions and thereby inhibit infection. To test this hypothesis, we preincubated cell-free preparations of HCMV strain TB40/E with variable concentrations of a soluble PDGFR-alpha-Fc chimera for two hours prior to infection of HFFs and a human endothelial cell line (HEC-LTTs; denoted as HECs). After another two hours, the virus was removed and replaced with the appropriate cell culture medium for an overnight incubation. Cultures were then fixed, and the fraction of infected cells was determined by indirect immunofluorescence staining of viral IE antigens. In fact, the PDGFR-alpha-Fc chimera inhibited infection of HFFs in a dose dependent manner with a half maximal effective concentration (EC50) of about 10–20 ng/ml and a complete abrogation of infection at 200 ng/ml (Fig 2, left panel). Unexpectedly, infection of HECs was also reduced, albeit at slightly higher concentrations (EC50 = 20–30 ng/ml) and inhibition was incomplete (Fig 2, right panel).

**Fig 3:**
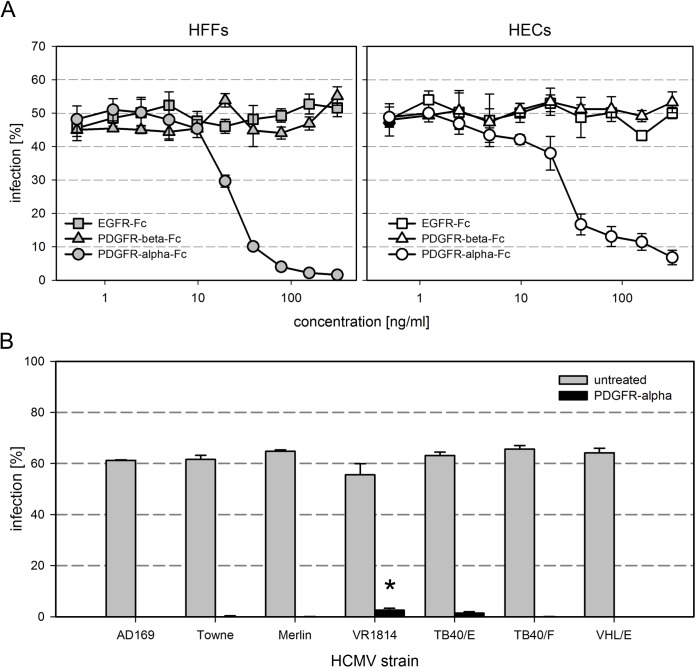
The inhibitory effect of soluble PDGFR-alpha is specific and affects various HCMV strains. **(A)** PDGFR-alpha-Fc, PDGFR-beta-Fc and EGFR-Fc were compared regarding their inhibitory potential on infection of fibroblasts (HFFs) and endothelial cells (HECs) by HCMV strain TB40/E. Virus preparations were pretreated for 2 h with the respective growth factor receptor at indicated concentrations and then added to cell cultures for 2 h, followed by a medium exchange and incubation overnight. Cells were fixed and stained for viral IE antigens. The percentage of infection was calculated as the ratio of IE antigen-positive cells / total cell number. **(B)** The potential of PDGFR-alpha-Fc to inhibit fibroblast infection with various HCMV strains was tested using a collection of strains representing the known glycoprotein variants. The virus preparations were either preincubated with medium (no drug) or medium containing 250 ng/ml PDGFR-alpha-Fc. Error bars in (A) and (B) represent the standard error of the mean (SEM). The significant difference of VR1814 as compared to the other strains is indicated by an asterisk.

To address the possibility that the effect is non-specifically mediated by the Fc part of the chimeric molecule, we compared PDGFR-alpha-Fc with PDGFR-beta-Fc and EGFR-Fc chimeras regarding their inhibitory potential on HCMV infection. Again, cell-free preparations of TB40/E were preincubated with increasing concentrations of the various Fc chimeras for two hours. HFFs and HECs were then incubated with the mixtures for 2 h followed by a medium exchange and an overnight incubation. Evaluation of the degree of infection by immunofluorescence staining of viral IE antigens showed that only PDGFR-alpha-Fc blocked infection in a dose dependent fashion, whereas neither PDGFR-beta-Fc nor EGFR-Fc had an effect (Fig 3A). As the Fc-part is identical with all three molecules, the growth factor receptor part of the PDGFR-alpha-Fc chimera is obviously required for the inhibitory effect.

Next, we wondered whether soluble PDGFR-alpha-Fc would inhibit not only strain TB40/E but also other HCMV strains. We therefore prepared cell-free stocks of TB40/E, its fibroblast-restricted variant TB40/F and five other HCMV strains (AD169, Towne, Merlin, VR1814, VHL/E) that represent different phylogenetic glycoprotein variants described for HCMV [38]. Virus preparations were pretreated for two hours with PDGFR-alpha-Fc at a concentration that was sufficient for complete inhibition of strain TB40/E in HFFs in the previous dose response experiments (250 ng/ml). The mixtures were added to HFFs and incubated overnight. The fraction of infected cells was determined by immunofluorescence staining of viral IE antigens. All strains were strongly inhibited by pretreatment with the soluble receptor (Fig 3B), and the reduction of infection was almost complete. Strains VR1814 and TB40/E showed a residual infection in 2.3% and 1.2% of cells, which only reached significance for VR1814 when compared to the other strains (p-values > 0.05). In conclusion, all tested strains were susceptible to inhibition by PDGFR-alpha-Fc irrespective of whether they contain the pentameric glycoprotein complex (VR1814, VHL/E, TB40/E) or not (AD169, Towne, Merlin, TB40/F).

Finally, to test whether this inhibitory effect was specific for HCMV we repeated the experiment and included another species of Herpesviridae, HSV-1 strain F. While the inhibitory effect on HCMV could be again reproduced, HSV infection was not affected by PDGFR-alpha-Fc (S2 Fig), indicating that the effect is specific for HCMV.

### Inhibition of HCMV infection by PDGFR-alpha-Fc occurs at the level of viral entry

The finding that pretreatment of virus with soluble PDGFR-alpha abrogated infection supported the idea of a direct physical interaction between PDGFR-alpha and virions. To test this, we aimed to detect bound PDGFR-alpha-Fc molecules on HCMV particles that have been pretreated with this molecule, using a fluorescence-labelled antibody against human IgG. Cell-free preparations of HCMV strain TB40/E were preincubated with PDGFR-alpha-Fc, PDGFR-beta-Fc or EGFR-Fc for two hours at 37°C. Virus particles were then allowed to attach to HFFs for 90 min on ice followed by fixation with acetone in order to immobilize them and to facilitate fluorescent staining. To visualize all virus particles, the abundant viral capsid-associated protein pp150 (pUL32) was stained in red via indirect immunofluorescence. Fc-fusion proteins were stained in green by direct immunofluorescence detecting the Fc part of the PDGFR-alpha-Fc fusion protein.

**Fig 4:**
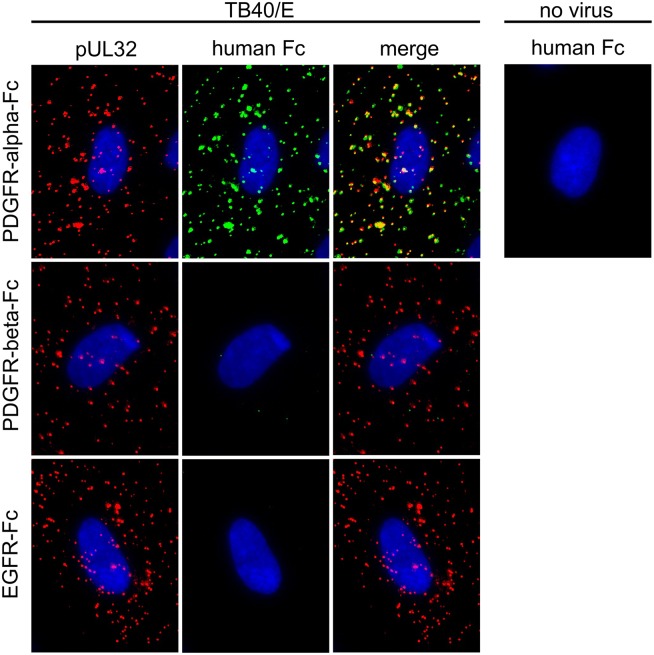
Binding of soluble growth factor receptor-Fc chimeras to HCMV particles. Virus preparations of strain TB40/E were pretreated for two hours with 500 ng/ml PDGFR-alpha-Fc, PDGFR-beta-Fc or EGFR-Fc and then incubated with fibroblasts for 90 min on ice. Cells were fixed and stained for the viral structural protein pUL32 (red) and for the Fc-fusion part (green). Nuclei were counterstained with DAPI.

Only after pretreatment with PDGFR-alpha-Fc, green fluorescence signals co-localized with attached virus particles whereas PDGFR-beta-Fc and EGFR-Fc did not yield signals (Fig 4). This is in accordance with the functional data regarding inhibition of infection and indicates that only PDGFR-alpha-Fc but not the other receptor-chimeras bind to HCMV particles. Mock-infected cells did not show any staining, proving that PDGFR-alpha-Fc did not bind to the cell surface in the absence of virus.

We then sought to further analyze the mode of action by comparing the dose-dependent binding of PDGFR-alpha-Fc with the dose-response curve for inhibition of infection. If all available binding sites contributed to infection (by attaching the virions to PDGFR-alpha on the cell surface) and inhibition was simply due to blocking of these binding sites, we expected both dose-response curves to be in the same concentration range. If binding of PDGFR-alpha-Fc not only blocks interaction sites but also induces alterations of virions, e.g. premature activation of fusion proteins, we expected inhibition already at concentrations far below saturation of binding sites. Similarly, the presence of different binding sites (e.g. active or inactive conformation of glycoproteins) might result in a difference between dose response curves for inhibition and binding.

**Fig 5:**
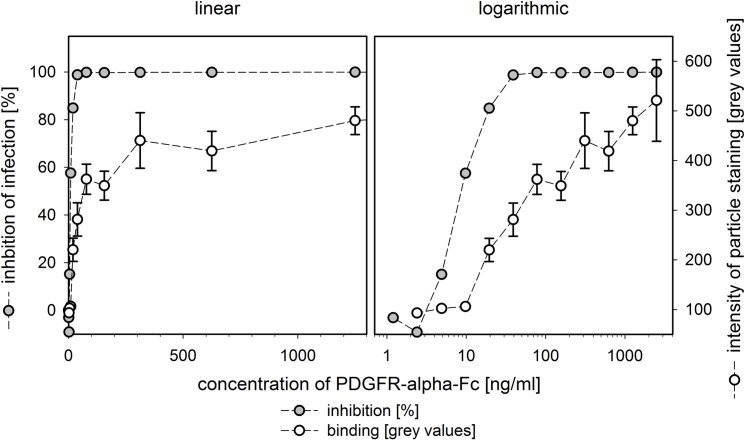
Quantification of PDGFR-alpha-Fc binding to HCMV particles. Virus preparations of strain TB40/E were preincubated with serial dilutions of PDGFR-alpha-Fc. Viruses were attached to fibroblasts by incubation on ice for 90 min followed by acetone fixation. Binding of PDGFR-alpha-Fc was assessed by direct immunofluorescence of the Fc-fusion part and quantification of signal intensities (maximum grey values per particle). In parallel, fibroblasts were incubated with the same mixtures at 37°C and stained for viral immediate-early antigens at one day postinfection to determine the fraction of infected cells. The intensity of the staining with PDGFR-alpha-Fc was measured for 100 particles in each condition. The same data set is provided with a linear scale and a logarithmic scale. Error bars indicate the error of the median.

Cell-free virus preparations were pretreated with a dilution series of PDGFR-alpha-Fc for two hours and virus particles were subsequently attached to HFFs on ice as described before. Again, attached particles were visualized by red staining of pUL32 and green staining of the Fc fusion part. Pictures were taken of both channels and a total of 100 viral particles per dilution was randomly selected according to the red pUL32 channel. For each particle, the maximum grey value of the Fc-staining was measured and median values representing the extent of binding at the respective concentration of PDGFR-alpha-Fc were calculated (Fig 5). In parallel, the same virus/PDGFR-alpha-Fc-mixtures were used to generate a dose-response curve of inhibition of infection as described above. Both curves converged towards a maximum (Fig 5, linear graph) but differed greatly regarding the half maximal concentration (Fig 5, logarithmic graph), with an EC50 for binding to HCMV particles of approximately 100 ng/ml while the EC50 for inhibition of HCMV was more than tenfold lower (approximately 10 ng/ml). This showed that PDGFR-alpha-Fc inhibits HCMV infection efficiently already at concentrations where only a minority of the total interaction sites are bound.

**Fig 6:**
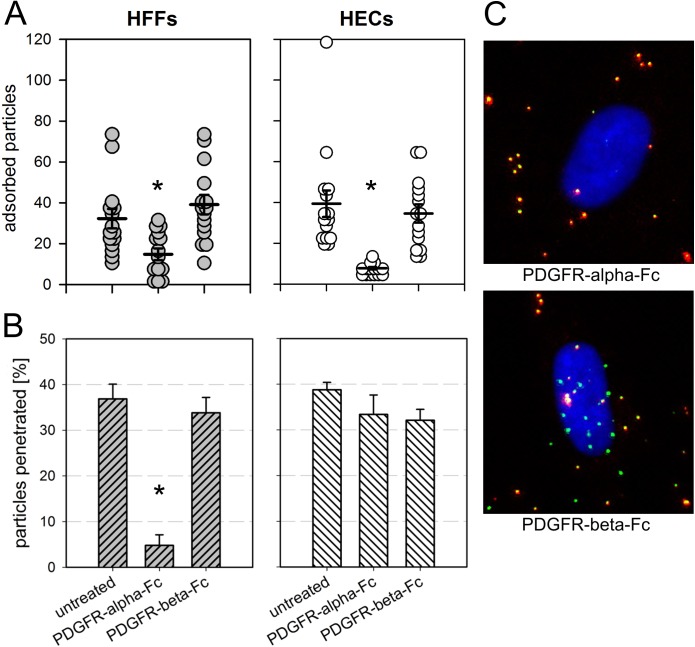
Effect of soluble PDGFR-alpha on adsorption and penetration of HCMV. Virus preparations of the dual fluorescent strain TB40-BAC_KL7_-UL32EGFP-UL100mCherry were preincubated with 100 ng/ml soluble Fc-chimeras for two hours and subsequently used to infect fibroblasts (HFFs) and endothelial cells (HECs) at 37°C for two hours. **(A)** Adsorption was assessed by counting the total number of bound virus particles per cell (pUL32-EGFP signals regardless of the pUL100-Cherry signals). Each dot represents one cell, mean values are indicated by a horizontal line, and error bars represent the standard error of the mean (SEM). Significant differences as compared to the untreated controls are indicated by asterisks. **(B)** The fraction of penetrated particles was determined by counting the percentage of particles lacking the envelope (particles without the pUL100-mCherry signal). Bars indicate the mean values of 15 cells per condition and error bars represent the standard error of the mean (SEM). One representative experiment out of three is shown. The significant difference between PDGFR-alpha-Fc-treated HFFs and the untreated control is indicated by an asterisk. **C**: Examples of microscopic images taken in HFFs. Enveloped virus particles appear yellow due to an overlap of pUL32-EGFP signals and pUL100-Cherry signals. Penetrated non-enveloped particles are only EGFP-positive. Nuclei were stained with DAPI.

To further investigate which of the initial steps of infection are blocked, we performed a series of experiments in HFFs and HECs to dissect adsorption and penetration. We used the dual fluorescent virus TB40-BAC_KL7_-UL32EGFP-UL100mCherry as it allows to discriminate between enveloped (both EGFP- and mCherry-positive) and non-enveloped (only EGFP-positive) particles [39]. We compared adsorption and penetration of untreated particles to particles preincubated with 100 ng/ml PDGFR-alpha-Fc or PDGFR-beta-Fc by counting the number of enveloped (= adsorbed, but not penetrated) versus non-enveloped (= penetrated) particles. On both cell types, adsorption of PDGFR-alpha-Fc-treated particles was reduced (by 50% on HFFs and 75% on HECs; Fig 6), indicating that soluble PDGFR-alpha-Fc generally hinders HCMV attachment. The reduction of binding was significant in both cell types (p-values < 0.05). In contrast, inhibition of penetration was cell type-specific, with PDGFR-alpha-Fc-treated particles penetrating HFFs 75% less efficiently than untreated controls (p-value < 0.05) whereas penetration was not specifically inhibited in HECs.

**Fig 7:**
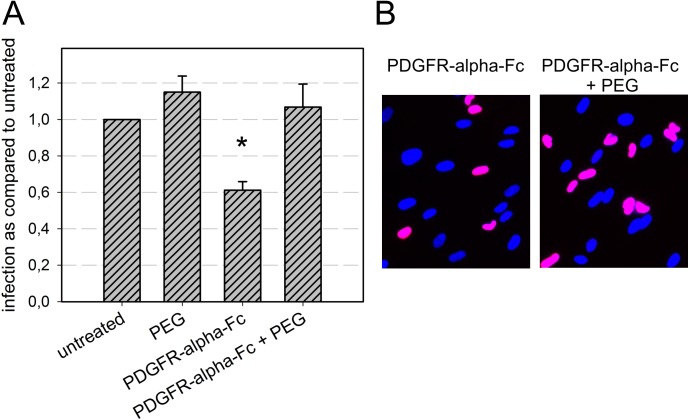
Postadsorption-inhibitory effect of soluble PDGFR-alpha-Fc. Virus preparations were adsorbed to fibroblasts on ice for 60 min and subsequently incubated with 200 ng/ml PDGFR-alpha-Fc for two hours on ice. Cells were then either directly shifted to 37°C or first treated with the chemical fusogen PEG. After an overnight incubation, cells were fixed and stained for viral IE antigens. **(A)** For each condition, the fraction of infected cells (immediate-early antigen positive cells/total cells) was compared to the untreated control. Mean values of 3 independent experiments are shown. Error bars indicate the standard error of the mean (SEM). The significant difference between PDGFR-alpha-Fc-treated cells and the untreated control is indicated by an asterisk. **(B)** Examples of HCMV IE antigen expression (red fluorescence) after treatment with PDGFR-alpha-Fc alone or PDGFR-alpha-Fc and PEG. Nuclei were counterstained with DAPI.

As these results indicated that pretreatment with PDGFR-alpha-Fc inhibits fusion of the viral envelope with cellular membranes in HFFs, we tested whether this inhibitor could also block entry of virus particles that have already attached to HFFs. HCMV virus particles were adsorbed to HFFs for 1 h on ice. The virus-containing medium was then exchanged by medium containing PDGFR-alpha-Fc at a concentration of 200 ng/ml. Inhibition of preadsorbed virus was performed for additional 2 h on ice, before the cells were shifted to 37°C to allow entry. After 2 h of incubation at 37°C, the cells were supplied with fresh medium without inhibitor and further incubated overnight. After 24 h, the cells were fixed and stained for the viral IE antigens. PDGFR-alpha-Fc reduced infectivity of already attached viruses to 60% and this reduction was significant (p-value < 0.05) (Fig 7).

To corroborate the idea that this postattachment-effect was due to inhibition of fusion, we tested whether the reduced fusion capability can be overcome by addition of the chemical fusogen polyethylene glycol (PEG). Therefore, cells with preadsorbed viruses were identically treated with PDGFR-alpha-Fc, but then PEG was added for 30 s before it was replaced with PDGFR-alpha-Fc containing medium for the 2 h incubation at 37°C. Postattachment inhibition was completely reversed by addition of PEG (p-value < 0.01), whereas PEG did not increase the infection efficiency of untreated control virus, suggesting that PDGFR-alpha-Fc actually inhibits the fusion step of HCMV entry under these experimental conditions. Alternatively, considering a recent report on endosomal uptake into fibroblasts [40], PDGFR-alpha-Fc might interfere with an endocytic process and thus inhibit the downstream fusion step.

**Fig 8:**
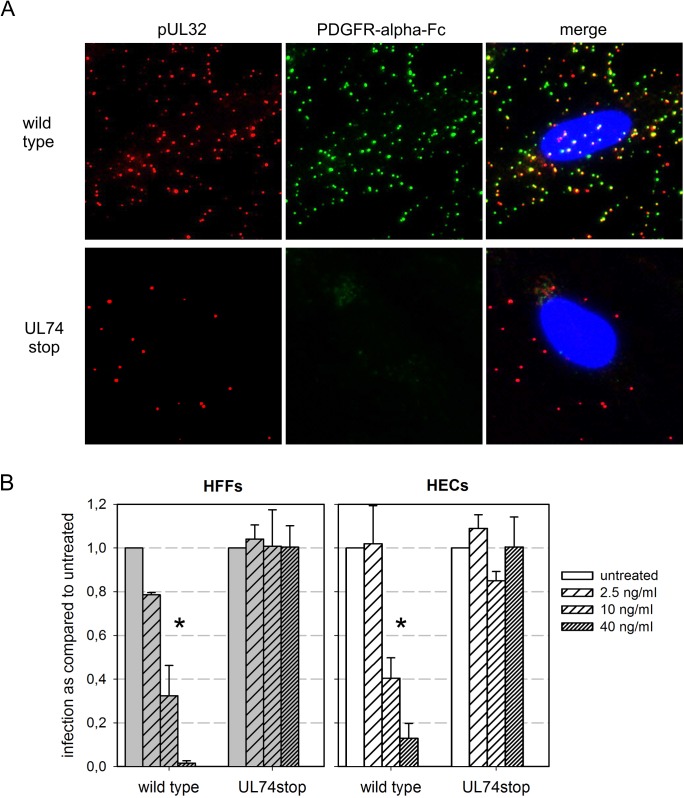
PDGFR-alpha-Fc binds HCMV particles only in the presence of the gH/gL/pUL74 complex. **(A)** Virus preparations of strain TB40-BAC4 (wild type) or TB40-BAC4-UL74stop were pretreated with 500 ng/ml PDGFR-alpha-Fc for two hours at 37°C. Fibroblasts (HFFs) were then incubated with pretreated virus on ice followed by immunofluorescence staining for the viral structural protein pUL32 (red) and the Fc-fusion part of PDGFR-alpha-Fc (green). **(B)** Virus preparations were preincubated with variable concentrations of PDGFR-alpha-Fc for 2h before infection of HFFs and endothelial cells (HECs). One day postinfection, cells were fixed and stained by immunofluorescence for viral immediate early antigens. For each condition, the fraction of infected cells (immediate-early antigen positive cells/total cells) was compared to the untreated control. Mean values of 3 independent experiments are shown. Error bars indicate the standard error of the mean (SEM). Significant differences as compared to the untreated controls are indicated by asterisks.

Regarding possible viral interaction partners of PDGFR-alpha, it is currently unclear whether it binds to gB (pUL55) [31] or gO (pUL74) [37] or both. We compared wild type HCMV particles with pUL74-deficient viral particles regarding their ability to bind PDGFR-alpha-Fc chimera (Fig 8), thereby enabling a discrimination between the two proposed interaction partners. If gB was the binding partner, PDGFR-alpha-Fc should bind to both wild type and mutant. If gO (pUL74) was the binding partner, it should bind only to wild type particles. One experimental challenge was posed by the fact that deletion of pUL74 strongly reduces infectivity, which is partially explained by a reduction of the ability of mutant virions to bind to cells (S3 Fig). Therefore, TB40-BAC4-UL74stop virus preparations had to be concentrated by ultracentrifugation in order to achieve sufficient particle numbers on cells for a comparison with wild type virus. Following this adjustment procedure, wild type or UL74stop particles were incubated with 500 ng/ml (i.e. a concentration that would yield saturated binding, see Fig 5) of PDGFR-alpha-Fc for two hours prior to attachment to the cells on ice. The particles on the cells were again visualized with an antibody recognizing the structural protein pUL32 and bound Fc-chimeras were stained with an Alexa488-conjugated anti-human-IgG antibody (Fig 8A). Only virus particles containing the glycoprotein pUL74 were stained with the fluorescent antibody against the Fc-part of the soluble receptor molecule, indicating that the trimeric gH/gL/pUL74 complex but not gB is involved in binding of PDGFR-alpha-Fc to virions.

To investigate whether lack of the trimeric complex not only abrogates binding but also renders virus insensitive to the inhibitory effect of PDGFR-alpha-Fc, UL74stop virus and wild type virus were compared regarding the PDGFR-alpha-Fc-mediated inhibition of infection in HFFs and HECs (Fig 8B; see also S4 Fig). As deletion of pUL74 from the virus greatly reduced infectivity of viral progeny, mutant virions had to be concentrated 50-fold by ultracentrifugation to achieve infection efficiencies up to 40% in HECs and 5% in HFFs. Wild type virus was used at dilutions that yielded a comparable level of infection. As expected, infection with wild type virus was significantly reduced by PDGFR-alpha-Fc in a dose-dependent fashion (p-values < 0.0001). In contrast, the infectivity of the UL74stop virus did not significantly change with increasing doses of PDGFR-alpha-Fc, indicating that the inhibitory effect of PDGFR-alpha-Fc is mediated via gH/gL/pUL74.

### Peptides derived from the extracellular domain of PDGFR-alpha inhibit HCMV infection

The finding that only PDGFR-alpha-Fc but not PDGFR-beta-Fc or EGFR-Fc inhibits HCMV infection indicated that the inhibitory effect is specific for the PDGFR-alpha part of the chimeric molecule. PDGFR-alpha-Fc contains only the extracellular domain of the native PDGFR-alpha transmembrane molecule. We hypothesized that peptides derived from this part of the protein could also inhibit infection. Therefore, we tested a set of overlapping 40mer peptides, which cover the whole sequence of the extracellular PDGFR-alpha domain, regarding their inhibitory potential. Cell-free preparations of the Gaussia luciferase-expressing HCMV strain TB40-BAC4-IE-GLuc were preincubated with the individual peptides for two hours at concentrations ranging from 0.05–50 nmol/ml. The mixtures were then used to infect HFFs and HECs for two hours at 37°C. Virus-Peptide mixtures were replaced with the appropriate growth medium and cells were further incubated overnight. Luciferase containing supernatants were harvested and luminescence indicating the extent of infection was counted in a plate reader using coelenterazine as a substrate. The degree of neutralization was calculated as 1- (luminescence / maximal luminescence).

**Fig 9:**
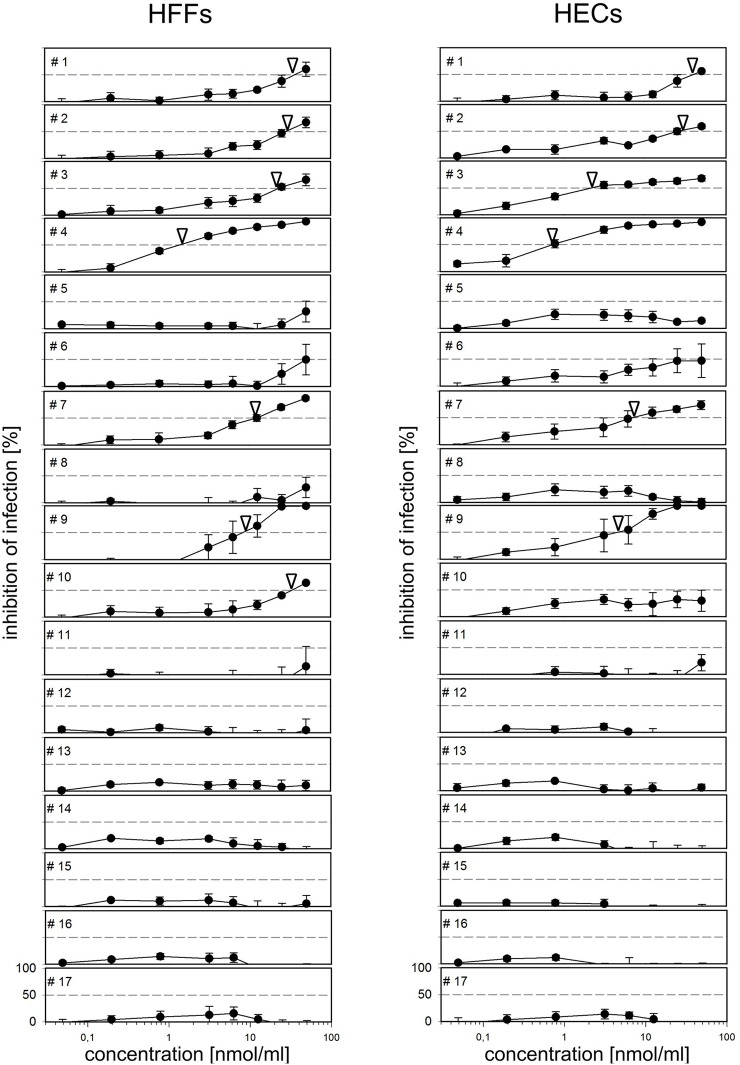
Inhibitory effect of PDGFR-alpha-derived peptides. The luciferase reporter virus TB40-BAC4-IE-Gluc was preincubated with 40mer peptides derived from the extracellular domain of PDGFR-alpha at concentrations from 0.05–50 nmol/ml for 2 h before infection of fibroblasts (HFFs) and endothelial cells (HECs). Untreated virus was used as a control for uninhibited infection. One day postinfection, supernatants were analyzed for luciferase activity indicating the degree of infection. The extent of inhibition was calculated as 1 - (luminescence with peptide / luminescence without peptide) and is given as percentage. Error bars indicate the standard error of the mean (SEM) of four data sets per peptide. The number of the peptide (#1-#17) indicates its position in the protein, starting from the N-terminus. Arrowheads indicate the concentration at which 50% of infection are inhibited.

The various peptides differed greatly regarding their inhibitory potential: the C-terminal region from peptide no 11 to peptide no 17 (corresponding to aa300-aa505) was ineffective (Fig 9). The peptide GT-40 (#4 in Fig 9), ranging from aa91 to aa130 (GRHIYIYVPDPDVAFVPLGMTDYLVIVEDDDSAIIPCRTT), was particularly effective with an EC50 of 2 nmol/ml and almost complete inhibition at 10 nmol/ml, irrespective of the cell type. Like the full-length protein, this peptide was also effective against other strains of HCMV (S6 Fig). At higher concentrations, some of the other peptides in the N-terminal part of the extracellular domain of PDGFR-alpha also inhibited infection in both cell types to some extent.

## Discussion

The finding that soluble platelet-derived growth factor receptor alpha binds to HCMV virions only if they contain the gH/gL/gO trimer, thereby inhibiting entry into fibroblasts and endothelial cells, has implications for our basic understanding of HCMV entry and may provide the starting point for a novel antiviral strategy.

PDGFR-alpha has repeatedly been reported as a cellular factor that can mediate HCMV infection [31, 32, 37]. However, for the first time we directly visualize the binding of PDGFR-alpha to virions of HCMV. The fact that binding is abrogated in virions of pUL74 deletion mutants, which lack the viral complex gH/gL/gO in their envelope, suggests that the trimer is the only viral interaction partner of the cell surface protein PDGFR-alpha. This finding is in line with a recent report showing that soluble gO can bind to this growth factor receptor [37]. As gO deletion mutants have been shown to contain normal amounts of gB in their envelope [41] our data directly argues against a binding to the envelope complex formed by gB, which has initially been suggested [31]. This fits with a previous report questioning a receptor-binding activity of gB [42].

Concerning EGFR, the fact that soluble EGFR-Fc did not inhibit infection in either cell type argues against a role for EGFR as a binding receptor on the cell surface, although it does not preclude a more indirect contribution of this molecule during HCMV entry, e.g. via signaling [43, 44].

While inhibition of fibroblast infection by pretreatment of virions with soluble PDGFR-alpha-Fc can be explained by interference with the binding of gH/gL/gO on virions to PDGFR-alpha on the cell surface, it appears surprising at a first glance that infection of endothelial cells was also inhibited by almost 90%. This seems to conflict with the fact that treatment of endothelial cells with PDGFR-alpha-specific siRNA had no effect on infection of endothelial cells, similar to previous findings in epithelial cells [32]. In support of the siRNA data, the lack of detectable PDGFR-alpha staining in our FACS analyses indicates that this receptor is not available for the promotion of HCMV entry into endothelial cells, which is concordant with available mRNA expression data from primary cell cultures (GEO data set accession #GDS1402). Hence, an interference of soluble PDGFR-alpha with infection of endothelial cells was not expected. On the other hand, Zhou et al. recently showed that the gH/gL/gO trimer is also necessary for efficient entry into endo- and epithelial cells [26]. Hence, it is conceivable that soluble PDGFR-alpha bound to the trimer might impede its penetration-promoting function. However, our mode-of-action analysis, in which only the binding step but not the subsequent penetration step was significantly reduced in endothelial cells, indicates that PDGFR-alpha-Fc does not interfere with this contribution of gH/gL/gO. Alternatively, PDGFR-alpha-Fc molecules that are bound to trimers in the virion envelope might sterically interfere with other interactions between HCMV and endothelial cells, e.g. binding of the pentamer to a yet undefined receptor. Regardless of the exact mode of action, the fact that PDGFR-alpha-Fc can block infection in both cell types obviously increases the potential to exploit this inhibitory molecule for antiviral treatment strategies.

One problem of current HCMV treatment options is that all approved drugs (ganciclovir, valganciclovir, foscarnet and cidofovir) target the same step of the viral life cycle, which is replication of the viral genome [1, 45]. They all frequently cause severe adverse effects including myelosuppression and nephrotoxicity, and this is particularly disadvantageous in transplant recipients with preexisting dysfunction of the kidney and/or the bone marrow. Furthermore, mutations have been reported that confer resistance to all of these drugs. Hence, alternative approaches are needed to provide treatment options in cases where the currently available drugs should be avoided. An inhibitor of the viral terminase that contributes to genome packaging is currently in a phase III trial [45]. This inhibitor causes little adverse effects but also induces resistance mutations [46, 47].

Another possible target for antiviral strategies is the beginning of the replicative cycle when HCMV must attach to target cells and fuse its envelope with a cellular membrane for entry into the cytoplasm. One well established way to interfere with these initial steps of viral infection is blocking of viral envelope proteins by neutralizing antibodies. In case of HCMV, the envelope glycoproteins gB, gH, and the accessory proteins of the pentameric gH/gL complex are major targets of neutralizing antibodies [6, 48, 49]. Remarkably, such neutralizing antibodies are only moderately effective against infection of fibroblasts as compared to their effect on epi- and endothelial cells [6, 8, 50, 51]. Entry into fibroblasts might be blocked specifically by antibodies against gO [37, 52], which are usually not found in HCMV-seropositive individuals, or by soluble PDGFR-alpha-derivatives. Based on our data with the UL74-deletion mutant, PDGFR-alpha-Fc can be assumed to specifically neutralize gO and thus act as a mimic of anti-gO antibodies. Noteworthy, the interaction of HCMV virions with cell surface receptors can be targeted in the extracellular space, thus greatly reducing the risk of interference with intracellular pathways and opening possibilities for molecules that cannot easily penetrate cells, like proteins and peptides.

Importantly, PDGFR-alpha-Fc inhibited all tested HCMV strains almost completely, including three strains that express both the trimer and the pentamer (VHL/E, TB40/E and VR1814). In a recent report, VR1814 was about 10-fold less sensitive to inhibition by soluble PDGFR-alpha than AD169 [37], which might suggest that this difference is due to expression of the pentamer. Our data argue against the assumption that the pentamer would render HCMV resistant against inhibition by PDGFR-alpha-derivatives, as VHL/E was completely inhibited and the residual infectivity with TB40/E was around 1% at an inhibitor concentration of 10 x EC50. Only VR1814 differed significantly from the other strains by a slightly higher residual infectivity, suggesting that strain-specific differences might exist independent from pentamer expression. As all three proteins of the trimer show polymorphisms, minor interstrain differences in the binding to PDGFR-alpha would not be too surprising. It is also noteworthy that the soluble PDGFR-alpha used by Kabanova et al. was more than a log step less efficient that the PDGFR-alpha-Fc molecule that we applied, which may indicate a contribution of the Fc part. In line with this interpretation, an anti-gO antibody was effective against VR1814 in both fibroblasts and epithelial cells [37], which fits perfectly with our finding that PDGFR-alpha-Fc is also effective in endothelial cells.

Regarding the distinct steps during viral entry that are inhibited, PDGFR-alpha-Fc did not reduce binding during incubation on ice (Fig 4 and S5 Fig) suggesting that the initial low affinity binding to heparan sulfate proteoglycans (HSPGs) is unaffected. In contrast, PDGFR-alpha-Fc reduced binding to both cell types during incubation at 37°C (Fig 6A). One possible explanation is that binding of PDGFR-alpha-Fc to the trimer cannot impede the initial attachment to HSPGs but interferes with a secondary high affinity binding to other cell surface molecules, e.g. receptors of the gH/gL-complexes. Unexpectedly, adsorption was also affected in endothelial cells although they lack PDGFR-alpha. One explanation is that trimer-bound PDGFR-alpha-Fc sterically hinders interaction of the pentamer with its (unknown) cellular receptor simply by increasing the distance between virion and cell surface. Another possibility is that the trimer itself is involved in binding of virions to endothelial cells via an unidentified receptor, which is supported by the unexpected finding that adsorption to endothelial cells is reduced with UL74stop virions (S3 Fig). At present, too little is known about the exact role of the trimer for entry into endothelial cells to favor one of these options. Finally, the penetration efficiency of adsorbed particles was only affected in fibroblasts but not in endothelial cells (Fig 6B). Taken together, it appears that binding of soluble PDGFR-alpha to virus particles inhibits the high affinity attachment to both cell types and in addition blocks fusion of the viral envelope with cellular membranes specifically in fibroblasts. Further support for an effect on fusion is provided by the finding that PDGFR-alpha-Fc blocks infection of HFFs even when applied after initial attachment and that this block can be overcome by the chemical fusogen PEG (Fig 7). The fact that two different steps of the entry process are inhibited in HFFs but only one step is affected in HECs may also explain the difference regarding the maximal effect that can be achieved with soluble PDGFR-alpha in the respective cell type. While inhibition in HFFs is complete, a small residual PDGFR-alpha-Fc-resistant infectivity remains in HECs (Figs 2, 3 and 8). It is tempting to speculate that this residual infection occurs via a pathway that is independent of the trimer and only relies on the pentamer. It has recently been reported that the trimer is necessary for efficient fusion in both cell types [26]. Our data support this conclusion but also suggest that there is a minor trimer-independent entry pathway in HECs whereas the trimer is almost indispensable in HFFs. This pathway is probably irrelevant during natural infection by wild type virus but becomes apparent when the trimer-mediated pathways are not available, as in case of the UL74stop mutant.

Somewhat unexpectedly, inhibition of infection is already complete at concentrations that saturate only a low proportion of possible binding sites (Fig 5). This may be explained by the presence of at least two different binding sites, with the higher affinity binding site contributing more to virus entry than the lower affinity binding site. Alternative explanations are that binding to only few of the trimeric complexes can trigger a premature inactivation of the fusion protein gB or sterically hinder interaction with the cell by preventing the binding of other active trimeric complexes to PDGFR-alpha on the cell surface. Concerning the therapeutic potential, this may be relevant because compounds that rely on complete saturation usually do not work for treatment of patients whereas compounds that are fully active at lower concentrations are more promising regarding the ratio of desired and adverse effects [53, 54]. In case of PDGFR-alpha-Fc, inhibition of HCMV already occurs at concentrations that are 10-100fold below those reported for binding to the natural ligands in the information of the reagent provider (R&D systems). If this applies also *in vivo*, an antiviral effect can be expected at doses that would not significantly bind and sequester the natural ligand, thus limiting unwanted effects. Due to its mode of action as an inhibitor of viral adsorption and penetration, which is analogous to neutralizing antibodies, PDGFR-alpha-Fc can only be expected to block cell-free virus transmission but will probably be ineffective regarding cell-to-cell spread.

A PDGFR-alpha-derived entry inhibitor would fall into the class of biologicals, which are in general characterized by their low toxicity [54]. This could be particularly advantageous, when considering treatment of pregnant women with primary infection, treatment of fetuses infected *in utero* and long-term treatment of congenitally infected newborns [2]. Remarkably, a similar approach has been successful in an animal model of coxsackie virus, where Fc-CAR was effective in preventing viral dissemination and disease [13, 14, 55]. Clinical experience with surface receptor-Fc chimeras is already available in rheumatic disease, where a tumor necrosis factor receptor-Fc fusion protein is well established in treatment regimens [56].

Beside the whole extracellular domain of PDGFR-alpha, small peptides derived from its sequence are an alternative therapeutic option. There is an increasing amount of evidence proving the excellent tolerability of host-derived peptides [57] and peptides have been shown to be effective particularly against viral entry [12, 58]. Hence, our finding that a PDGFR-alpha-derived 40mer can also efficiently reduce infection in both cell types at a concentration of 3–30 nmol/ml provides a promising starting point for further optimization [54]. The fact that inhibition achieved with this peptide (95% in HFFs) was lower than with PDGFR-alpha-Fc (99% in HFFs) could for example be due to a lower affinity of the peptide as compared to the complete extracellular domain of the receptor, and this indicates that further improvements might be possible.

Considering their therapeutic application, both PDGFR-alpha-Fc and PDGFR-alpha-derived peptides may offer a number of advantages: (i) they are completely host-derived and therefore assumed to be non-immunogenic, (ii) an additive effect with the established anti-HCMV drugs can be expected due to the different modes of action; (iii) in contrast to most antibodies [5, 8] they are almost equally effective against infection via the pentamer-dependent and the pentamer-independent entry pathway and (iv) resistance-conferring mutations would most likely affect the entry potential of the virus and hence reduce viral fitness. In conclusion, soluble derivatives of PDGFR-alpha can effectively inhibit entry of HCMV into various cell types, which might pave the way for the development of a therapeutic entry inhibitor.

## Methods

### Cells and viruses

Primary human foreskin fibroblast (HFFs) were isolated from tissue samples that were residuals from routine procedures. Samples were obtained anonymized after written informed consent of the parents in agreement with articles 21 and 23 of the recommendations of the council of Europe (2006). HFFs were propagated in MEM supplemented with GlutaMAX (Life Technologies), 5% fetal calf serum (FCS; PAN Biotech), 100 μg/ml gentamicin and 0.5 ng/ml basic fibroblast growth factor (bFGF; Life technologies). Experiments were carried out in HFF-medium without bFGF (denoted as MEM5). Conditionally immortalized human endothelial cells (HEC-LTT, denoted as HECs), were kindly provided by D. Wirth [59, 60]. HEC-LTTs are human umbilical vein endothelial cells (HUVECs) that contain doxycycline-controlled expression cassettes for the human telomerase catalytic subunit (hTERT) and the simian virus 40 large T-antigen (SV40-TAg) [59]. In the presence of the doxycycline, hTERT and SV40-TAg expression are activated, resulting in high cell proliferation and unlimited expansion. HECs were cultured in gelatin-coated vessels using endothelial cell growth medium (EGM bullet kit; Lonza) supplemented with 2 μg/ml doxycycline. For infection experiments, HECs were seeded the day before in the absence of doxycycline, resulting in a growth-arrested state resembling primary HUVECs. The efficiently transfectable and HCMV-susceptible hybrid endothelial cell line EA.hy926 (ATCC CRL-2922) [60–62] was expanded in DMEM (life technologies) supplemented with 10% FCS.

The HCMV strain TB40 has been previously isolated in our lab from a bone marrow transplant patient [63]. The highly endotheliotropic version TB40/E was the result of long-term propagation on endothelial cells whereas TB40/F lost the pentameric complex during cultivation on fibroblasts and is therefore non-endotheliotropic. AD169 [64], Towne [65] and Merlin [66, 67] are widely used HCMV strains that also lack the pentameric complex. VR1814 [68], VHL/E [69] represent endotheliotropic HCMV strains. TB40-BAC_KL7_-UL32EGFP-UL100mCherry is a BAC-cloned derivative of TB40/E that was fluorescently labelled to allow differentiation between enveloped and non-enveloped virus capsids [39]. TB40-BAC4 is a highly endotheliotropic BAC-clone based on TB40/E [70] and served as the basis for mutant BAC4-UL74stop which lacks the expression of pUL74 (gO) [71]. The reporter virus TB40-BAC4-IE-GLuc contains a Gaussia luciferase expression cassette under control of the major immediate-early enhancer/promoter at an ectopic position [72].

Infectious supernatants of TB40 variants, AD169, Towne, VHL/E and VR1814 were harvested from infected HFFs at five to seven days postinfection. Supernatants were cleared from cells and cellular debris by centrifugation at 2,700 x g for 10 min before storage at -80°C. Cleared UL74stop supernatants were 50-fold concentrated by ultracentrifugation at 70,000 x g for 70 min. Virus stocks of TB40-BAC4-IE-GLuc were first cleared from cellular debris as described above and virions were washed twice by ultracentrifugation at 70,000 x g for 70 min to minimize background levels of secreted luciferase.

### Chimeric receptor molecules and PDGFR-alpha-derived peptides

All recombinant Fc-fusion proteins used in this study were obtained from R&D: PDGFR-alpha-Fc, PDGFR-beta-Fc and EGFR-Fc. Proteins were dissolved in phosphate buffered saline (PBS) at a concentration of 500μg/ml.

The 40mer peptides based on the extracellular domain of human PDGFR-alpha isotype 1 were obtained from Phtdpeptides (Shanghai, China) at a purity of > 95%. Depending on their physiochemical properties, peptides were dissolved in either water, 0.1 M ammonium carbonate, 10% acetic acid or dimethyl sulfoxide to a concentration of 1 mmol/l.

### Determination of infection efficiencies

Infection efficiencies were determined by indirect immunofluorescence staining of viral IE proteins pUL122/123. Cells were fixed with 80% acetone for 5 min and incubated sequentially with primary mouse antibody E13 (Argene) and secondary antibody Cy3-goat anti-mouse IgG F(ab′)2 (Jackson ImmunoResearch). To locate nuclei, cells were counterstained with 4′,6-diamidino-2-phenylindole (DAPI). The percentage of infection was determined by counting the number of IE-positive cells, as well as the total number of nuclei per image. For each condition, three images were evaluated.

To determine infection efficiencies with the reporter virus TB40-BAC4-IE-GLuc, a 20 μl aliquot of each cell culture supernatant was transferred to a 96-well luminescence reader plate. 50 μl of the substrate coelenterazine (0.2 μg/ml; PjK) were injected using a microplate reader with a built-in injection system (Chameleon, Hidex) and luminescence was measured. For all obtained values, background luminescence was subtracted and neutralization efficiencies were calculated.

### Depletion of protein expression by siRNA

For reverse transfection of siRNAs, pools of four different siRNAs (siGENOME Dharmacon) per target were complexed with Lipofectamine RNAiMAX (Life Technologies) in 96-well plates. HFFs and EA.hy926 cells were added at a density of 10,000 cells per well. A highly efficient HCMV-IE siRNA [62] (Sigma-Aldrich) served as a positive control and the siGENOME non-targeting pool #2 (Dharmacon) was used as a negative control. Duplicate wells of all conditions were prepared. Two days posttransfection, cells were infected with HCMV-TB40/E at a multiplicity of infection (MOI) 1. The next day, cells were fixed with 80% acetone and stained for viral IE antigens.

### Fluorescence-activated cell sorting

To analyze the effect of siRNA-mediated knockdown on cell surface expression of PDGFR-alpha and EGFR, HFFs and EA.hy926 cells were transfected with the respective siRNA or non-targeting siRNA, harvested 2 d posttransfection, and incubated on ice with PE-conjugated anti-PDGFR-alpha-antibody (FAB1264P, R&D), Fluorescein-conjugated anti-EGFR-antibody (FAB10951F, R&D) or the respective isotype controls. Cells were then washed twice in PBS with 2% FCS and analyzed in a FACS Calibur (BD Biosciences). The obtained data sets were processed with Flowing Software version 2.5.1 (by Perttu Terho).

### Inhibition of infection

For testing the inhibitory effect of Fc-fusion proteins or peptides on HCMV, agents were diluted in MEM5 and mixed with virus preparations at a concentration resulting in a final MOI of 1. The mixtures were incubated for 2 h at 37°C before addition to the cells. HFFs and HECs seeded on gelatin-coated 96-well plates at a density of 1.5 x 10^4^ cells per well were preincubated with MEM5 for 30 min, medium was exchanged against virus mixtures and incubated for 2 h. Virus mixtures were removed and replaced by the respective growth medium. Cells were incubated overnight and infection efficiencies were measured as described above. EC50 values were determined by non-linear regression applying sigmoidal dose response curve fitting (Sigma Plot).

### Binding of chimeric receptors to HCMV particles

To assess the binding of Fc-Proteins to virus particles, HFFs were seeded on gelatin-coated 8-well μ-slides (Ibidi) at a density of 4 x 10^4^ cells per well one day prior to infection. Virus preparations were preincubated with Fc-fusion proteins at indicated concentrations for 2 h at 37°C and subsequently precooled on ice for 15 min. Medium of precooled cells was exchanged against virus mixtures and incubated on ice for 90 min. The cells were washed once with MEM5 prior to fixation with 80% acetone. For staining of viral particles, cells were incubated with a mouse monoclonal antibody recognizing the abundant viral protein pUL32 (generously provided by W. Britt) [73]. As a secondary antibody, Cy3-conjugated goat anti-mouse IgG F(ab’)_2_ (Jackson ImmunoResearch) was used. Visualization of bound Fc-proteins was achieved by applying Alexa488-conjugated goat-anti-human IgG (H+L; Invitrogen). For better orientation, cell nuclei were stained with DAPI. For quantification of PDGFR-alpha-Fc binding to HCMV particles, the grey values of 100 particles per condition were analyzed using AxioVision Software (Zeiss).

### Quantification of adsorption and penetration

HFFs and HECs were seeded on gelatin-coated 8-well μ-slide (Ibidi) at a density of 4 x 10^4^ cells per well. Freshly produced cell-free infectious supernatant of TB40-BAC_KL7_-UL32EGFP-UL100mCherry was mixed with MEM5 (untreated control) or MEM5 with 100 ng/ml soluble Fc-chimeras and incubated for 2 h at 37°C. Following a preincubation of the cells with MEM5 for 30 min, medium was exchanged against virus mixtures and incubated for 20 min at 37°C. Virus mixtures were exchanged against MEM5 (untreated control) or MEM5 with 100 ng/ml of the respective Fc-chimera and incubated for another 100 min at 37°C. Cells were fixed with 80% acetone for 5 min and sequentially reacted with anti-GFP mouse IgG_2a_ (clone 3E6, Invitrogen), Alexa Fluor488-conjugated goat anti-mouse IgG F(ab′)_2_ fragments (Invitrogen) and DAPI. Pictures of each individual channel (native red fluorescent signals; stained green pUL32-EGFP signals and blue nuclei) were taken and particles of a total of 15 cells per condition were counted.

### Analysis of postadsorption inhibitory effects

HFFs were seeded on two gelatin-coated 8-well μ-slides (Ibidi) at a density of 4 x 10^4^ cells per well. Cells were precooled on ice for 15 min and medium was subsequently exchanged against ice-cold, freshly produced cell-free infectious supernatant of TB40/E. Attachment of viral particles was allowed on ice for 1 h. Supernatant was exchanged against precooled MEM5 with or without 200 ng/ml PDGFR-alpha-Fc and incubated on ice for 2 h. One of the plates was shifted to 37°C for 2 h, whereas cells of the replica-plate were treated with prewarmed 50% polyethylene glycol 1500 PEG; Roche) for 30 sec. The PEG was immediately removed by five-times washing with prewarmed PBS and cells were incubated in prewarmed MEM5 with or without 200 ng/ml PDGFR-alpha-Fc for 2 h at 37°C. Medium was exchanged to MEM5 on both replica plates and infection was allowed to proceed for 24 h before infection efficiencies were assessed by staining of viral IE antigens.

### Statistical analyses

Datasets were analyzed by one-way-ANOVA using the build-in data analyses function of Excel to test whether there are significant differences between the various conditions. If ANOVA indicated significant differences between groups within the data set, post-hoc analyses were performed to identify which of the groups differ from others. Depending on the structure of the data sets, post-hoc analyses were done by t-test (Figs 1 and 3), Dunnett's test (Fig 6) or Tukey test (Fig 7), using the build-in data analyses functions of Excel (t-test) or Sigmaplot (Dunnett’s, Tukey). Only the p-values of post-hoc tests are mentioned in the results section. The error of the median (Fig 5) was determined by 1000fold resampling (bootstrapping) from our data sets of 100 cells per condition.

## Supporting information

S1 Fig Effects of treatment with siRNAs specific for PDGFR-alpha and EGFR. (A) In order to test the efficiency of the siRNA-mediated depletion, surface expression of PDGFR-alpha was visualized by immunofluorescence. HFFs and endothelial hybrid cells (Ea.hy926) were transfected with pools of non-targeting (NT) siRNA and siRNA directed against PDGFR-alpha in 96-well μ-clear plates. Two days after transfection, cells were precooled on ice and subsequently incubated with monoclonal antibodies against PDGFR-alpha (clone 35248; ThermoFisher) for 90 min on ice before fixation with 80% acetone. The primary antibody was detected with Cy3-conjugated goat polyclonal anti-mouse Ig F(ab')2 antibody. Cell nuclei were counterstained with DAPI. (B): Fluorescence-activated cell sorting (FACS) for detection of EGFR on the surface of fibroblasts and endothelial cells 2 d after treatment with EGFR siRNA or non-targeting (NT) siRNA. NT siRNA represents EGFR levels without specific knockdown. Isotype control antibody was included as a negative control for staining. (C) Fibroblasts and endothelial cells were transfected with siRNAs targeting EGFR. Non-targeting (NT) siRNAs and siRNAs against the viral immediate early (IE) proteins were included as controls. Two days after transfection, cells were infected with HCMV strain TB40/E, and the next day viral IE antigens were detected by indirect immunofluorescence for visualization of infected cells. The number of IE antigen-positive cells was counted and compared to the NT control. Error bars represent the standard error of the mean (SEM).
(TIF)

S2 Fig The soluble PDGFR-alpha-Fc has no inhibitory effect on HSV-1. In order to determine whether soluble growth factor receptor molecules inhibit HSV-1 infection, PDGFR-alpha-Fc, PDGFR-beta-Fc and EGFR-Fc were preincubated for 2 h at the indicated concentrations with HSV-1 strain F [1,2]. The mixtures were added to HFFs in duplicate wells and incubated for 1 h followed by a medium exchange. Cells were fixed 6 hours after infection with 80% acetone and stained for viral ICP0 antigen with a mouse anti-ICP0 antibody (clone 11060, Santa Cruz Biotechnologies). The primary antibody was detected with AF488-goat-anti mouse Ig-F(ab’)_2_ (Life Technologies) and the nuclei were counterstained with DAPI. The percentage of infected cells was calculated as the ratio of ICP0 antigen-positive cells / total cell number. The graph integrates data from two independent experiments. Error bars represent the standard error of the mean (SEM). Inhibition of HCMV, included as a positive control, was complete with a mean EC50 of 14 ng/ml (see Fig 2).
(TIF)

S3 Fig The adsorption of virions lacking pUL74 is impaired. Virions of TB40-BAC4 wild type and TB40-BAC4-UL74stop were gradient purified through glycerol-tartrate gradients [3]. One part of each preparation was used to detect adsorption whereas another portion was used to quantify the input virions. Adsorption of virus particles to HFFs and HECs was allowed for 1 h at 37°C before fixation with 80% acetone. Virus particles were visualized by staining for the capsid-associated tegument protein pUL32 and cell nuclei were counterstained with DAPI. The number of adsorbed virus particles was counted for about 60 cells per condition in each experiment. To determine the amount of input virions, the samples were treated with Qiagen RNase-free DNase for 30 min to remove viral DNA that is not protected within a capsid. After isolation of the DNA from the virions (using QIAamp Blood Mini Kit; Qiagen), the number of viral genomes was quantified by real-time PCR as described previously [4]. For calculation of the adsorption efficiencies of the different viruses, the number of input virions was compared to the number of adsorbed particles. Shown is the mean of three independent experiments and the standard error of the mean (SEM). The difference between wild type virus and UL74stop virus was highly significant in both cell types (p-value in HFFs < 0.01; p-value in HECs: < 0.01).
(TIF)

S4 Fig Infection of UL74stop virus is not inhibited by PDGFR-alpha-Fc. In order to achieve similar percentages of infection in the untreated samples, UL74stop virus was concentrated by ultracentrifugation whereas wild type virus was diluted. Virus preparations were preincubated with various dilutions of PDGFR-alpha-Fc for 2h before infection of HFFs and endothelial cells (HECs). The percentage of infected cells was determined one day post infection by calculation of the number of immediate-early positive nuclei over total DAPI stained nuclei per image. One representative experiment is shown.
(TIF)

S5 Fig Adsorption to fibroblasts on ice is not inhibited by PDGFR-alpha-Fc. TB40/E was pretreated for two hours with 500 ng/ml PDGFR-alpha-Fc or PDGFR-beta-Fc and then incubated with fibroblasts for 90 min on ice. After fixation with 80% acetone, virus particles were visualized by staining for the viral structural protein pUL32. Nuclei were counterstained with DAPI. The number of adsorbed particles/cell was determined for 25 cells per condition. Each dot represents one cell and the median is indicated by a horizontal line.
(TIF)

S6 Fig The PDGFR-alpha-derived peptide GT40 inhibits various HCMV strains. The potential of peptide GT40 to inhibit infection of fibroblast with various HCMV strains was tested using a collection of strains other than TB40/E. The virus preparations were preincubated either with medium (untreated control) or with medium containing peptide GT40 at 3 nmol/ml. Cells were fixed 1 d after infection, viral immediate early antigens were stained by indirect immunofluorescence, and the number of infected cells were compared in treated and untreated cultures. The reduction of infectivity is shown as percentage of the infectivity with untreated virus. Error bars represent the standard error of the mean (SEM). All strains were inhibited by peptide GT40 albeit to different levels (ranging from 47% to 89%).
(TIF)

S1 References Supporting references. (DOCX)
