# Supplementary figures and images for: A derivative of platelet-derived growth factor receptor alpha binds to the trimer of human cytomegalovirus and inhibits entry into fibroblasts and endothelial cells
Source: PLoS Pathog. 2017 Apr 12;13(4):e1006273. doi: 10.1371/journal.ppat.1006273 (PMC5389858; doi:10.1371/journal.ppat.1006273)

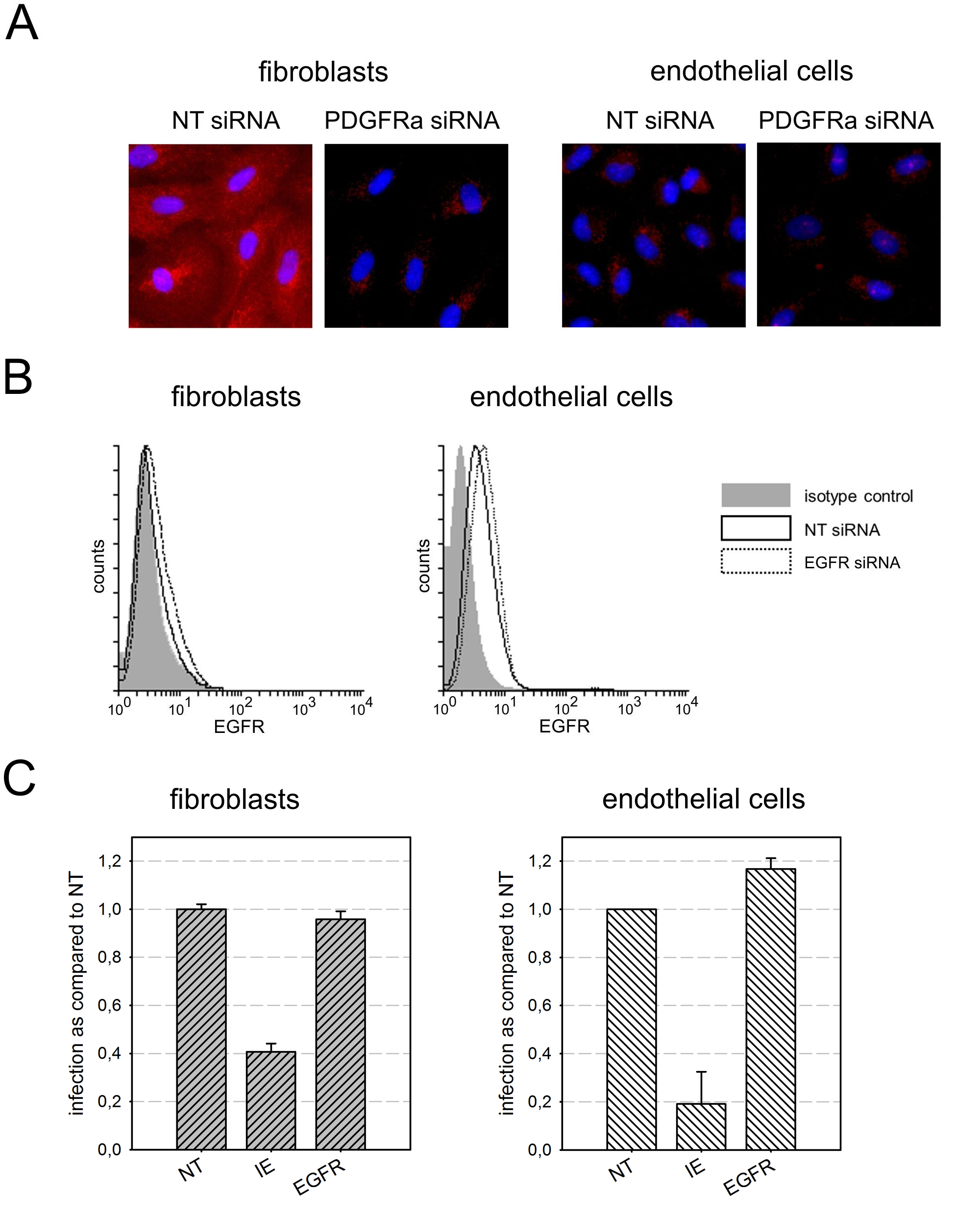

Supplement: S1 Fig — (A) In order to test the efficiency of the siRNA-mediated depletion, surface expression of PDGFR-alpha was visualized by immunofluorescence. HFFs and endothelial hybrid cells (Ea.hy926) were transfected with pools of non-targeting (NT) siRNA and siRNA directed against PDGFR-alpha in 96-well μ-clear plates. Two days after transfection, cells were precooled on ice and subsequently incubated with monoclonal antibodies against PDGFR-alpha (clone 35248; ThermoFisher) for 90 min on ice before fixation with 80% acetone. The primary antibody was detected with Cy3-conjugated goat polyclonal anti-mouse Ig F(ab')2 antibody. Cell nuclei were counterstained with DAPI. (B): Fluorescence-activated cell sorting (FACS) for detection of EGFR on the surface of fibroblasts and endothelial cells 2 d after treatment with EGFR siRNA or non-targeting (NT) siRNA. NT siRNA represents EGFR levels without specific knockdown. Isotype control antibody was included as a negative control for staining. (C) Fibroblasts and endothelial cells were transfected with siRNAs targeting EGFR. Non-targeting (NT) siRNAs and siRNAs against the viral immediate early (IE) proteins were included as controls. Two days after transfection, cells were infected with HCMV strain TB40/E, and the next day viral IE antigens were detected by indirect immunofluorescence for visualization of infected cells. The number of IE antigen-positive cells was counted and compared to the NT control. Error bars represent the standard error of the mean (SEM). (TIF) [file ppat.1006273.s001.tif]

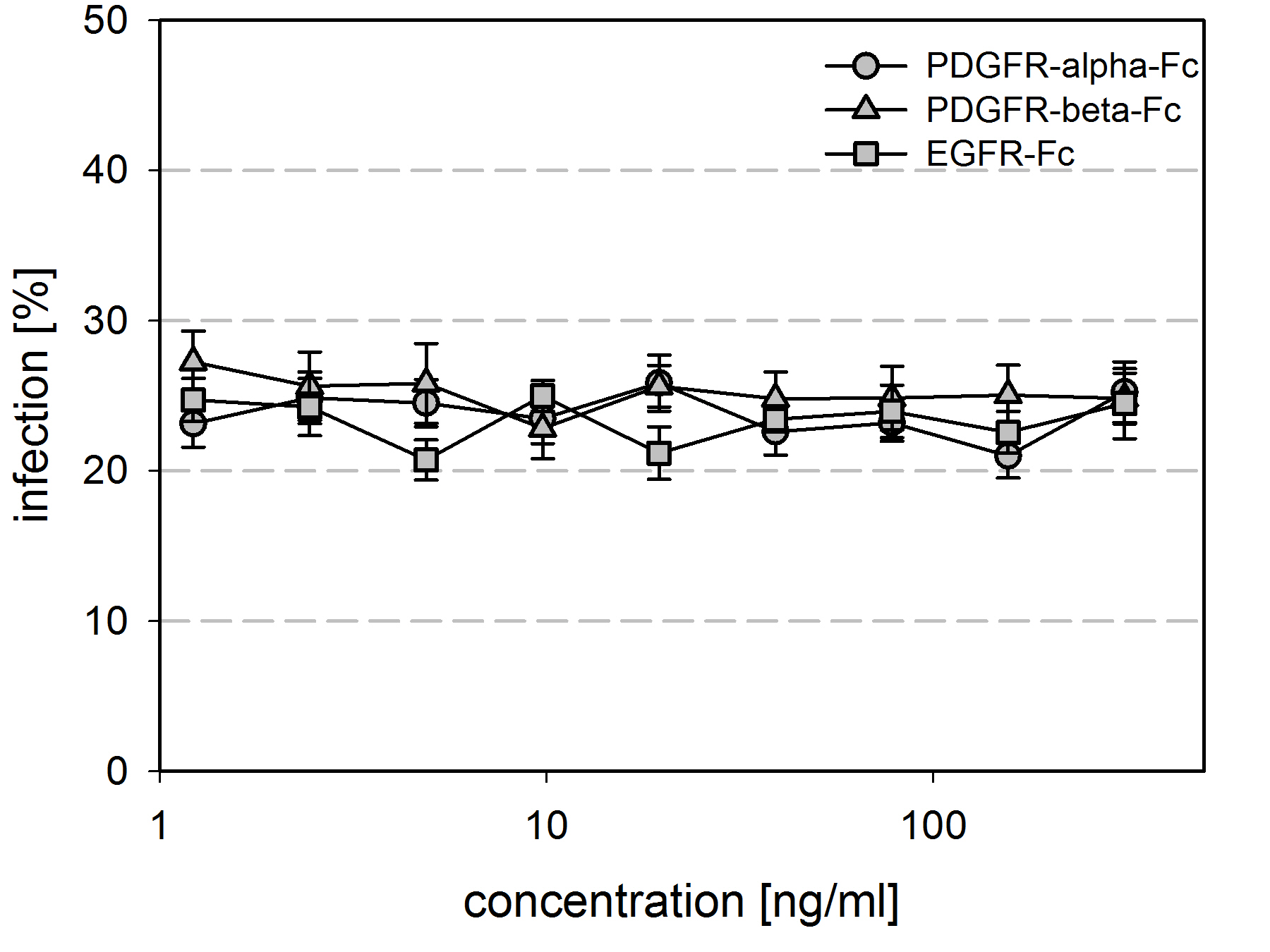

Supplement: S2 Fig — In order to determine whether soluble growth factor receptor molecules inhibit HSV-1 infection, PDGFR-alpha-Fc, PDGFR-beta-Fc and EGFR-Fc were preincubated for 2 h at the indicated concentrations with HSV-1 strain F [1,2]. The mixtures were added to HFFs in duplicate wells and incubated for 1 h followed by a medium exchange. Cells were fixed 6 hours after infection with 80% acetone and stained for viral ICP0 antigen with a mouse anti-ICP0 antibody (clone 11060, Santa Cruz Biotechnologies). The primary antibody was detected with AF488-goat-anti mouse Ig-F(ab’)2 (Life Technologies) and the nuclei were counterstained with DAPI. The percentage of infected cells was calculated as the ratio of ICP0 antigen-positive cells / total cell number. The graph integrates data from two independent experiments. Error bars represent the standard error of the mean (SEM). Inhibition of HCMV, included as a positive control, was complete with a mean EC50 of 14 ng/ml (see Fig 2). (TIF) [file ppat.1006273.s002.tif]

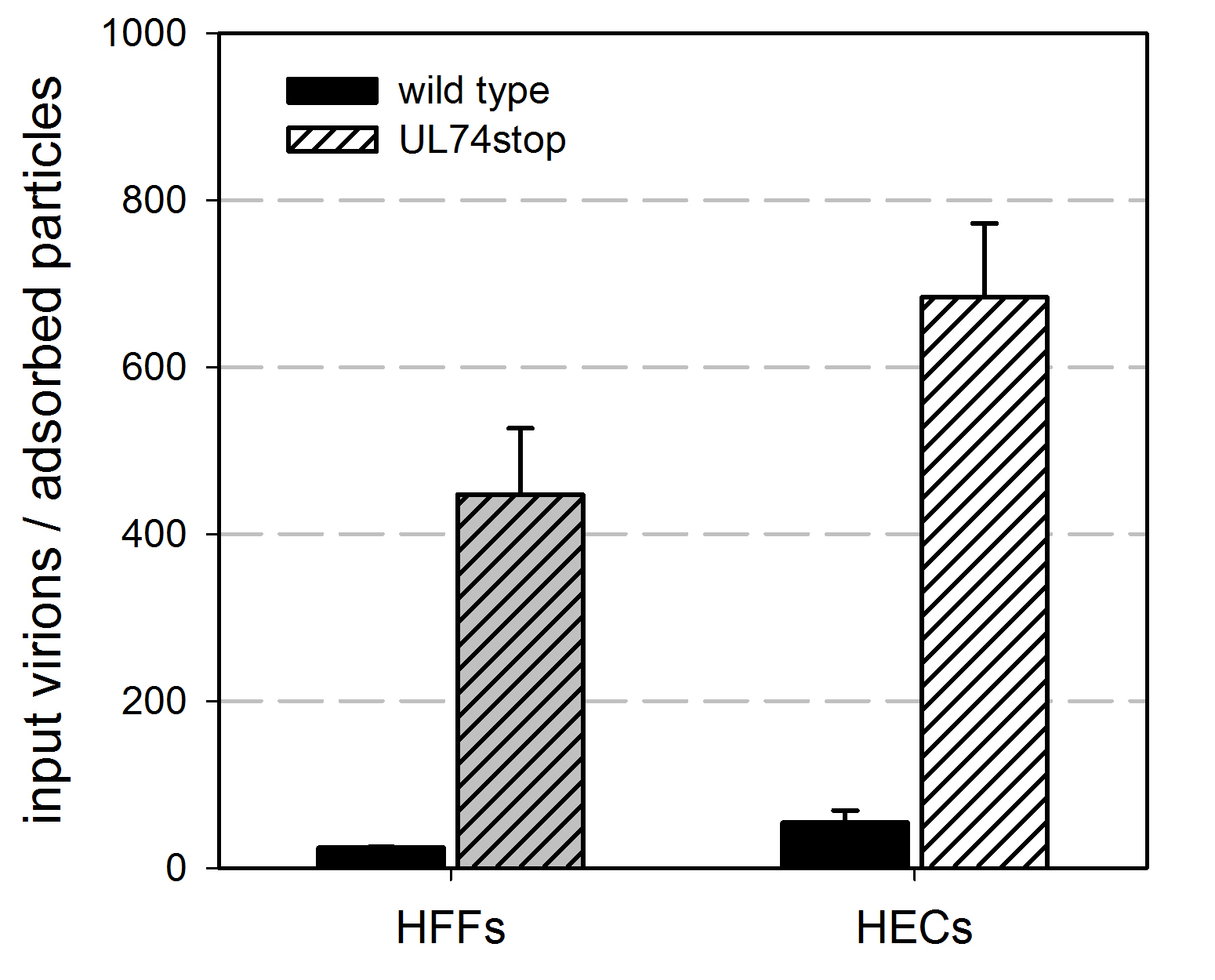

Supplement: S3 Fig — Virions of TB40-BAC4 wild type and TB40-BAC4-UL74stop were gradient purified through glycerol-tartrate gradients [3]. One part of each preparation was used to detect adsorption whereas another portion was used to quantify the input virions. Adsorption of virus particles to HFFs and HECs was allowed for 1 h at 37°C before fixation with 80% acetone. Virus particles were visualized by staining for the capsid-associated tegument protein pUL32 and cell nuclei were counterstained with DAPI. The number of adsorbed virus particles was counted for about 60 cells per condition in each experiment. To determine the amount of input virions, the samples were treated with Qiagen RNase-free DNase for 30 min to remove viral DNA that is not protected within a capsid. After isolation of the DNA from the virions (using QIAamp Blood Mini Kit; Qiagen), the number of viral genomes was quantified by real-time PCR as described previously [4]. For calculation of the adsorption efficiencies of the different viruses, the number of input virions was compared to the number of adsorbed particles. Shown is the mean of three independent experiments and the standard error of the mean (SEM). The difference between wild type virus and UL74stop virus was highly significant in both cell types (p-value in HFFs < 0.01; p-value in HECs: < 0.01). (TIF) [file ppat.1006273.s003.tif]

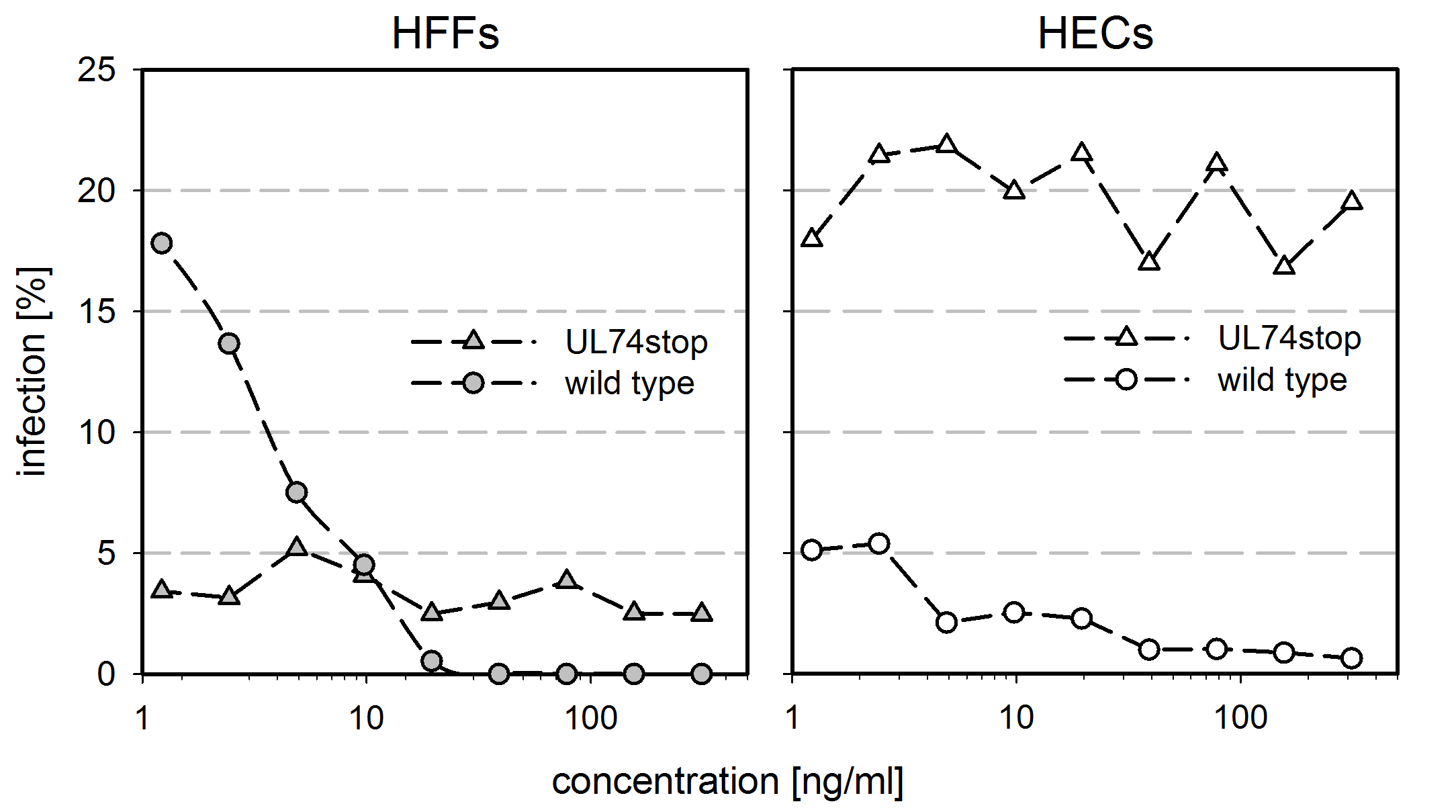

Supplement: S4 Fig — In order to achieve similar percentages of infection in the untreated samples, UL74stop virus was concentrated by ultracentrifugation whereas wild type virus was diluted. Virus preparations were preincubated with various dilutions of PDGFR-alpha-Fc for 2h before infection of HFFs and endothelial cells (HECs). The percentage of infected cells was determined one day post infection by calculation of the number of immediate-early positive nuclei over total DAPI stained nuclei per image. One representative experiment is shown. (TIF) [file ppat.1006273.s004.tif]

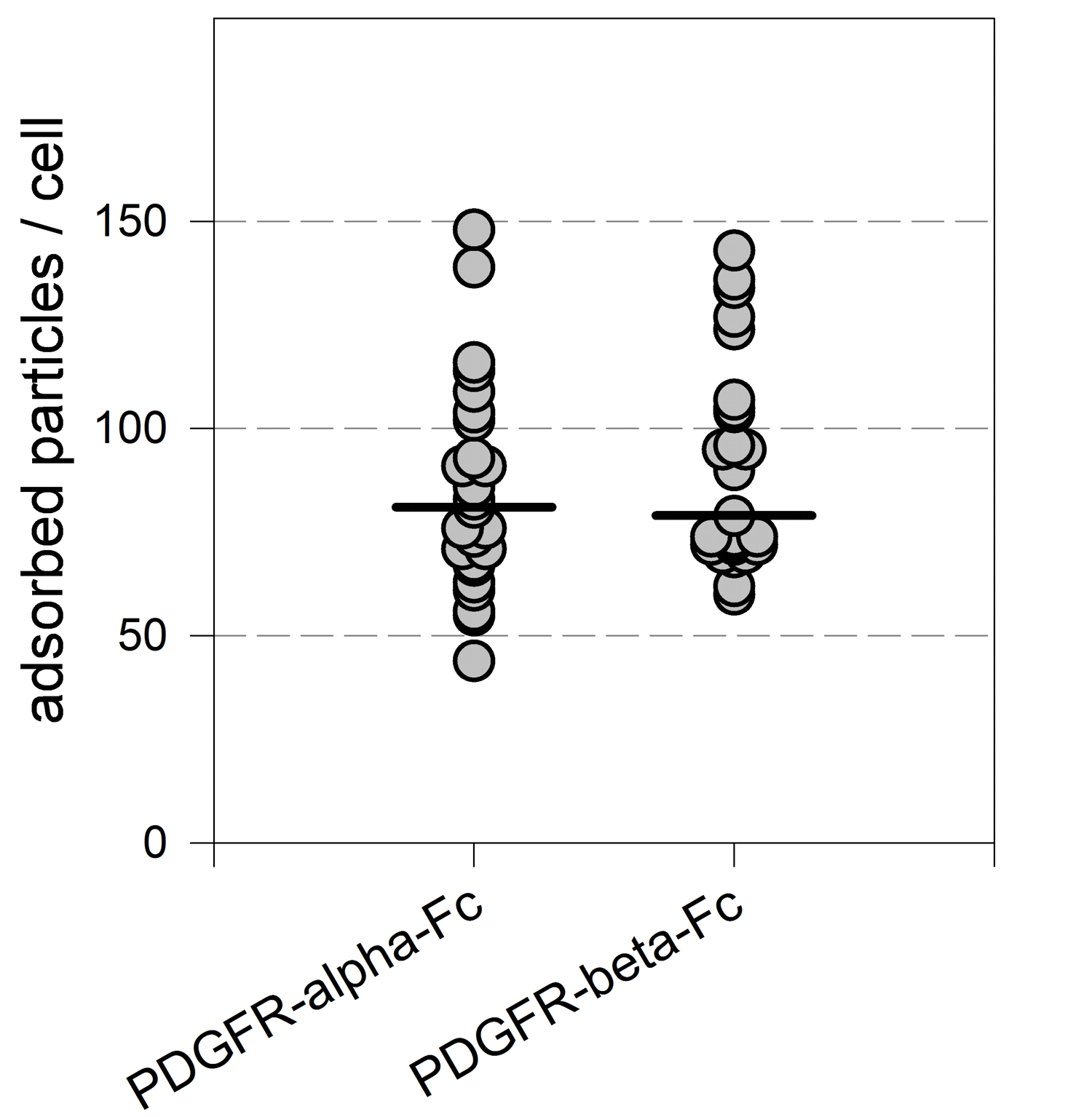

Supplement: S5 Fig — TB40/E was pretreated for two hours with 500 ng/ml PDGFR-alpha-Fc or PDGFR-beta-Fc and then incubated with fibroblasts for 90 min on ice. After fixation with 80% acetone, virus particles were visualized by staining for the viral structural protein pUL32. Nuclei were counterstained with DAPI. The number of adsorbed particles/cell was determined for 25 cells per condition. Each dot represents one cell and the median is indicated by a horizontal line. (TIF) [file ppat.1006273.s005.tif]

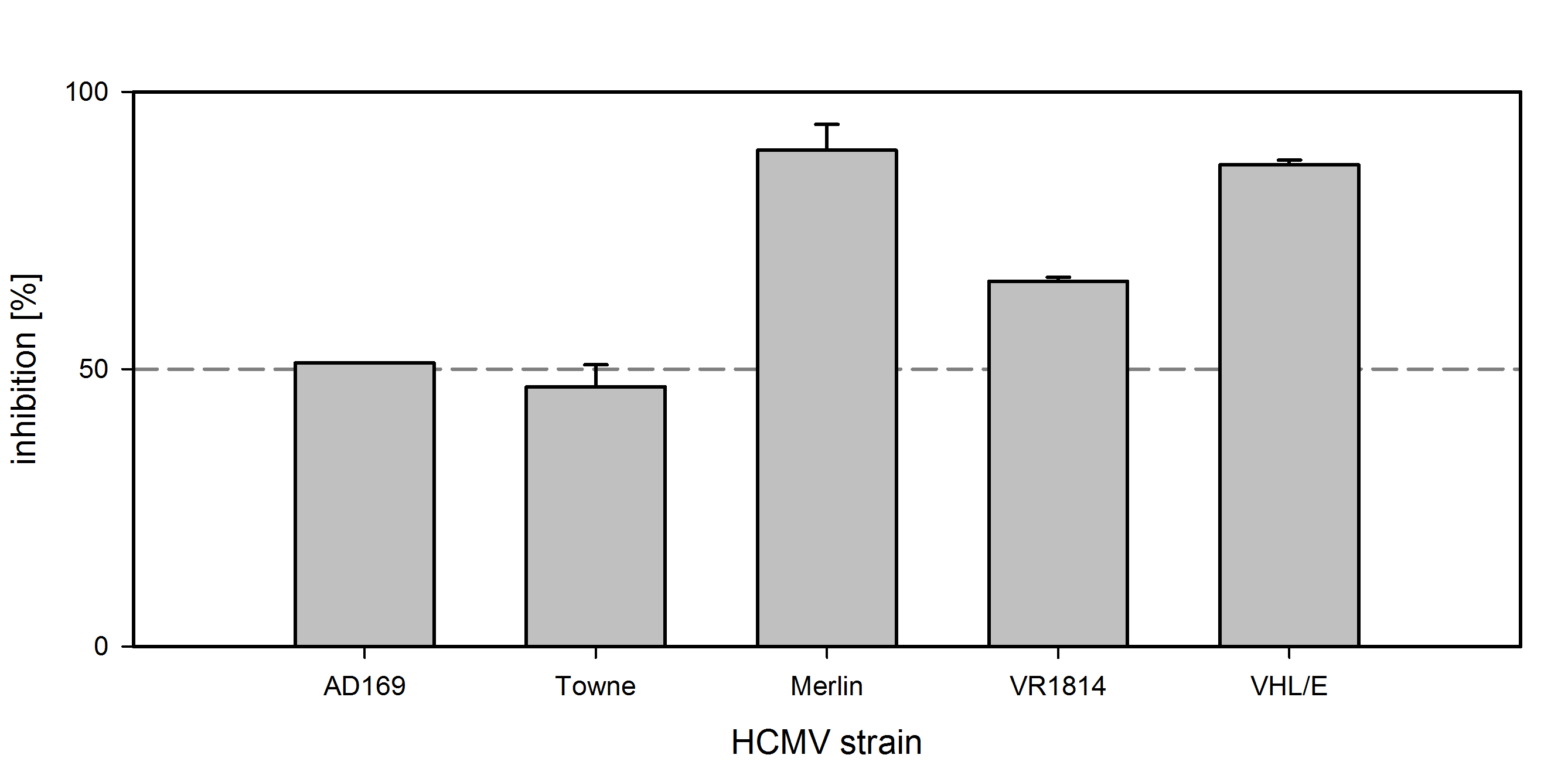

Supplement: S6 Fig — The potential of peptide GT40 to inhibit infection of fibroblast with various HCMV strains was tested using a collection of strains other than TB40/E. The virus preparations were preincubated either with medium (untreated control) or with medium containing peptide GT40 at 3 nmol/ml. Cells were fixed 1 d after infection, viral immediate early antigens were stained by indirect immunofluorescence, and the number of infected cells were compared in treated and untreated cultures. The reduction of infectivity is shown as percentage of the infectivity with untreated virus. Error bars represent the standard error of the mean (SEM). All strains were inhibited by peptide GT40 albeit to different levels (ranging from 47% to 89%). (TIF) [file ppat.1006273.s006.tif]
