# Supplementary material for: A derivative of platelet-derived growth factor receptor alpha binds to the trimer of human cytomegalovirus and inhibits entry into fibroblasts and endothelial cells
Source: PLoS Pathog. 2017 Apr 12;13(4):e1006273. doi: 10.1371/journal.ppat.1006273 (PMC5389858; doi:10.1371/journal.ppat.1006273)
Supplement: S1 References — (DOCX) [file ppat.1006273.s007.docx]

# References S1:

1. Baines JD, Koyama AH, Huang T, Roizman B. The UL21 gene products of herpes simplex virus 1 are dispensable for growth in cultured cells. J Virol. 1994 May;68(5):2929-36.

2. Ejercito PM, Kieff ED, Roizman B. Characterization of herpes simplex virus strains differing in their effects on social behaviour of infected cells. J Gen Virol. 1968 May;2(3):357-64.

3. Talbot P, Almeida JD. Human cytomegalovirus: purification of enveloped virions and dense bodies. J Gen Virol. 1977 Aug;36(2):345-9.

4. Sassenscheidt J, Rohayem J, Illmer T, Bandt D. Detection of beta-herpesviruses in allogenic stem cell recipients by quantitative real-time PCR. Journal of virological methods. 2006 Dec;138(1-2):40-8.
